# PCR-based specific techniques used for detecting the most important pathogens on strawberry: a systematic review

**DOI:** 10.1186/2046-4053-4-9

**Published:** 2015-01-15

**Authors:** Seyed Mahyar Mirmajlessi, Marialaura Destefanis, Richard Alexander Gottsberger, Marika Mänd, Evelin Loit

**Affiliations:** Department of Field Crops and Grassland Husbandry, Institute of Agricultural and Environmental Sciences, Estonian University of Life Sciences, Tartu, Estonia; Pesticides, Plant Health and Seed Testing Laboratories, Department of Agriculture, Food and the Marine, Backweston Campus, Celbridge, Co. Kildare Ireland; Department for Molecular Diagnostics of Plant Diseases, Institute for Sustainable Plant Production, Austrian Agency for Health and Food Safety (AGES), Vienna, Austria; Department of Plant Protection, Institute of Agricultural and Environmental Sciences, Estonian University of Life Sciences, Tartu, Estonia

**Keywords:** Molecular diagnostic methods, PCR-based techniques, Strawberry pathogen detection, Systematic review

## Abstract

**Background:**

Strawberry diseases are a major limiting factor that severely impact plant agronomic performance. Regarding limitations of traditional techniques for detection of pathogens, researchers have developed specific DNA-based tests as sensitive and specific techniques. The aim of this review is to provide an overview of polymerase chain reaction (PCR)-based methods used for detection or quantification of the most widespread strawberry pathogens, such as *Fusarium oxysporum* f.sp. *fragariae*, *Phytophthora fragariae*, *Colletotrichum acutatum*, *Verticillium dahliae*, *Botrytis cinerea*, *Macrophomina phaseolina*, and *Xanthomonas fragariae*. An updated and detailed list of published PCR protocols is presented and discussed, aimed at facilitating access to information that could be particularly useful for diagnostic laboratories in order to develop a rapid, cost-effective, and reliable monitoring technique.

**Methods:**

The study design was a systematic review of PCR-based techniques used for detection and quantification of strawberry pathogens. Using appropriate subject headings, AGRICOLA, AGRIS, BASE, Biological Abstracts, CAB Abstracts, Google Scholar, Scopus, Web of Knowledge, and SpringerLink databases were searched from their inception up to April 2014. Two assessors independently reviewed the titles, abstracts, and full articles of all identified citations. Selected articles were included if one of the mentioned strawberry pathogens was investigated based on PCR methods, and a summary of pre-analytical requirements for PCR was provided.

**Results:**

A total of 259 titles and abstracts were reviewed, of which 22 full texts met all the inclusion criteria. Our systematic review identified ten different protocols for *X. fragariae*, eight for *P. fragariae*, four for *B. cinerea*, six for *C. acutatum*, three for *V. dahlia*, and only one for *F. oxysporum*. The accuracy and sensitivity of PCR diagnostic methods is the focus of most studies included in this review. However, a large proportion of errors in laboratories occur in the pre-analytical phase of the testing process. Due to heterogeneity, results could not be meta-analyzed.

**Conclusions:**

From a systematic review of the currently available published literature, effective detection assays to detect the major strawberry pathogens have been developed. These assays can function as a basis for clinical labs, regulatory personnel, and other diagnosticians to adapt or implement for detection of these six important strawberry pathogens.

**Electronic supplementary material:**

The online version of this article (doi:10.1186/2046-4053-4-9) contains supplementary material, which is available to authorized users.

## Background

Strawberry (*Fragaria* × *ananassa*) is one of the world’s most commercially important fruit crops [1]. It was estimated that the global strawberry production in 2012 was 4,516,810 tons, according to Food and Agriculture Organization (FAO) statistics [2]. Strawberry diseases are a major limiting factor that severely impact the plant agronomic performance and lead to economic losses. Moreover, most strawberry cultivars are highly susceptible to several destructive and economically important pathogenic fungi and bacteria such as *Fusarium oxysporum* f.sp. *fragariae*, *Phytophthora fragariae*, *Colletotrichum acutatum*, *Verticillium dahliae*, *Botrytis cinerea*, *Macrophomina phaseolina*, and *Xanthomonas fragariae* [3, 4].

Early, rapid, and specific detection and identification of plant pathogens is essential for effective plant disease management [5]. Without specific disease diagnosis, proper control measures cannot be used at the appropriate time [6]. Conventional methods to detect and identify pathogens have often relied on isolating the pathogen onto selective media or through biochemical, chemical, and immunological analyses [7]. These methods are fundamental to diagnose the presence of plant pathogens, but they rely on time-consuming and labor-intensive lab techniques and on skilled taxonomical expertise [8]. Molecular-based techniques can overcome many of the shortcomings of the conventional assays, especially if they rely on polymerase chain reaction (PCR) assays. PCR-based assays are generally more specific and much faster than conventional techniques [5, 9]. Moreover, these techniques can also be applied on non-culturable microorganisms, as the organism does not need to be isolated to be identified by PCR [10]. An increasing amount of diagnostic methods recommended by the European and Mediterranean Plant Protection Organization (EPPO) are based on PCR assays [11, 12]. This technique is nowadays considered a routine technique in molecular diagnosis.

Plant disease management necessitates the need to reduce the spread of the pathogen. The extent in the optimum implementation of control strategies depends not only on the presence of a pathogen but also on the pathogen inoculum load. Thus, the capability of quantifying the pathogen load represents an important aspect of plant disease management [13]. Quantification based on culturing techniques is considered relatively nonspecific, while quantification using PCR techniques, in particular real-time PCR (rtPCR), provides a reliable estimation of the pathogen load. Unlike end-point PCRs, rtPCRs allow the detection of amplification products while the reaction is taking place, i.e., during each PCR cycle. Template quantification is highly specific because it assesses during the exponential phase of the reaction [9, 14]. Nowadays, a wide range of plant pathogens can be detected and quantified by PCR-based methods in numerous hosts or environmental samples [11, 15].

The necessity of fast, sensitive, and specific methods to detect pathogen is important to improve decision making in disease control. So, the primary objective of this review is to provide an exhaustive overview of the existing scientific literature available on PCR-based diagnostic techniques that is restricted to the detection and quantification of the seven most abundant strawberry pathogens: *F. oxysporum* f.sp. *fragariae*, *P. fragariae*, *C. acutatum*, *V. dahliae*, *B. cinerea*, *M. phaseolina*, and *X. fragariae*. A secondary objective is to determine the pre-analytical requirements of PCR assays (such as sample preparation of target pathogens and treatments prior to amplification). Finally, this compilation intends to provide an updated list of published PCR protocols for detection and quantification of strawberry pathogens with the aim of establishing a common diagnostic PCR based-method for routine testing by looking at the factors that affect the efficiency of the different test formats and comparing their performance in pathogen detection in plant material and soil.

## Methods

### Search strategy

In line with our experimental design, only relevant scientific papers published any time before 1 April 2014 and in the English language in a peer-reviewed journal were taken into consideration. The search was extended to libraries, such as AGRICOLA, AGRIS, BASE, Biological Abstracts, CAB Abstracts, Google Scholar, Scopus, Web of Knowledge, Science Direct, and SpringerLink, using the following identifiers: “PCR”, “molecular diagnostic”, “*F. oxysporum* f.sp. *fragariae*”, “*P. fragaria*”, “*C. acutatum*”, “*V. dahliae*”, “*B. cinerea*”, “*M. phaseolina*”, and “*X. fragariae*”. All associated terms were combined using “OR” and then “AND” to yield a total number of abstracts for each database (see Additional file 1). Two assessors (SMM, EL) independently reviewed the titles and abstracts of all identified citations. Results were limited to strawberry pathogens. Searches were carried out in all fields by default, and, where possible, searches were not restricted to titles or abstracts, but extended to the full text of the article. Both reviewers independently evaluated each full-text article. Disagreements were resolved by consensus. Table 1 lists all selected references that were included in the systematic review.

**Table 1:** **Studies included in the systematic review of PCR techniques used for detecting of strawberry pathogens**

Year	First author	Pathogen	PCR method	Sample preparation (long-term storage)	Origin of culture	Reference
1996	Sreenivasaprasad	CA	Conventional	NG	UK	[73]
1996	Roberts	XF	Conventional + nested	-70°C in 15% glycerol	US	[49]
1996	Pooler	XF	Multiplex	NG	US	[47]
1997	Bonants	PF	Nested	V8 oatmeal agar containing 50 ppm vancomycin/French bean agar at 4°C	Scotland + Netherlands	[45]
1997	Mahuku	XF	Nested	-70°C in 25% glycerol	Canada	[50]
1997	Zhang	XF	Conventional	NG	US	[74]
2002	Rigotti	BC	Conventional (Southern blot hybridization)	NG	Switzerland	[22]
2004	Stöger	XF	Conventional	NG	Austria	[16]
2004	Zimmermann	XF	Nested	-20°C in 30% glycerol	Germany	[48]
2004	Bonants	PF	Nested + real-time (TaqMan, Mol. Beacon) + PCR-ELISA	V8 agar at 11°C	Netherlands	[13]
2005	Suarez	BC	Real-time (TaqMan)	Frozen plastic bag at -20°C	UK	[21]
2006	Ioos	PF	Conventional	NG	France	[19]
2006	Drenth	PF	Conventional	Freeze-dried at -70°C	Australia	[72]
2007	Weller	XF	Real-time (TaqMan)	NG	UK	[51]
2008	Vandroemme	XF	Real-time (TaqMan)	NG	Belgium	[18]
2008	Turechek	XF	Real-time (TaqMan)	NG	US	[17]
2008	Pérez-Hernández	CA	Nested + conventional	NG	US	[29]
2008	Kuchta	VD	Conventional	Czapek-Dox Agar at 4°C	Poland	[46]
2009	Debode	CA	Real-time (TaqMan)	NG	Belgium	[27]
2009	Garrido	CA	Conventional + real-time (TaqMan)	Sterile water at 4°C	Spain + UK	[28]
2012	Bilodeau	VD	Multiplexed real-time (TaqMan)	NG	US	[33]
2013	Suga	FO	Multiplex	-80°C in 50% glycerol	Japan	[1]

*XF Xanthomonas fragariae*, *PF Phytophthora fragariae*, *BC Botrytis cinerea*, *VD Verticillium dahliae*, *FO Fusarium oxysporum*, *CA Colletotrichum acutatum*, *NG* not given.

### Selection criteria and data extraction

Titles and abstracts of papers detected using the search strategy described above were further examined in order to include only articles that investigate molecular diagnostic methods on strawberry pathogens. Thereafter, the articles were further selected if (1) the methods reported a summary of pre-analytical requirements for PCR and (2) the investigation included PCR methods applied on either of the following pathogens: *X. fragariae*, *P. fragariae*, *M. phaseolina*, *F. oxysporum*, *V. dahliae*, *B. cinerea*, and *C. acutatum*. Studies that described PCR-based diagnostic methods but did not investigate strawberry pathogens were excluded from the systematic review.

In addition to this, all references of the selected articles were scanned if the title of the article mentioned the use of molecular diagnostic methods on strawberry pathogens. The newly selected article underwent the same selection criteria outlined above. Four experts on the subject were identified from relevant publications and were contacted by email in order to receive advice on relevant literature on the molecular diagnostic methods in strawberry pathogens. Two responses were received. These included three articles, one was considered not relevant (based on the criteria outlined above) and the remaining two articles had been already identified in the preliminary search. Gray literature (conference abstracts and unpublished studies) and duplicate publications of the same data were disregarded.

### Study design and quality

The full text of all selected articles was read, and relevant information was extracted, summarized, and schematically outlined in tables. All methods described in the included articles were summarized in six tables (see supplemental information: Additional file 2: Tables S1, S2, S3, S4, S5, and S6) based on the aforementioned pathogens. They were referred to in the text by number (*S***#**). Moreover, each method included in the supplementary tables was assessed for quality on the basis of three criteria that were defined *a priori* as essential to answer the research questions: used PCR-based methods for detection and quantification of important pathogens on strawberry and soil samples, compared available methods through detection sensitivity and specificity of each method, and presented pre-analytical requirements (i.e., sample preparation) related to the accuracy of each method. Studies were defined satisfactory if they met all three criteria.

### Data analyses

The Results section focuses on important strawberry pathogens found in this review. The molecular methods used and main outcomes in each study were investigated. However, a statistical meta-analysis was not justified because of the heterogeneity of the included studies in detecting strawberry pathogens based on PCR-based assays. We synthesized the results (in the supplementary tables) according to PCR protocol, primer sets and target DNA employed in each study, pathogen treatment, and sensitivity of detection.

## Results

**Figure 1:**
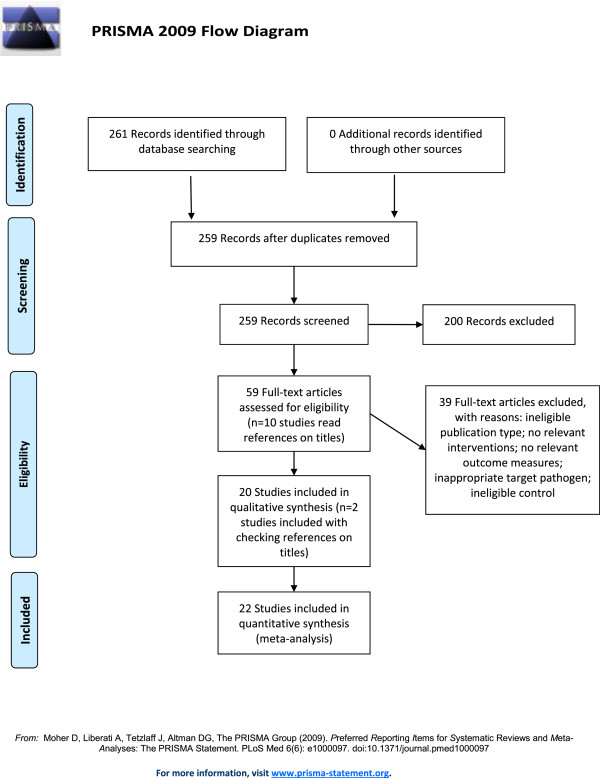
**Flow diagram of the study selection process for the systematic review.**

The articles originated from 1996 to 2013, with a rapid increase in the number of publications on the topic since 2004. The original systematic search strategy identified 259 unique citations, of which 200 articles were excluded based on the content of the title and/or abstract (Figure 1).

The initial search resulted in 259 hits. Fifty-nine were selected based on the title and abstract. Full text was read and references were checked for additional hits. This resulted in ten additional hits. Twenty-two papers were included based on the full text.

Fifty-nine articles were read and evaluated for inclusion criteria. This resulted in the inclusion of 20 articles. Ten articles were read based on references, of which two were included, bringing the sum of included relevant articles to 22 (Table 1). Several PCR-based techniques were investigated in the 22 selected articles: Nested PCR (nPCR) was investigated in six studies, real-time PCR (rtPCR) was adopted in 12 articles, conventional PCR (cPCR) was reported in ten articles, and four studies focused with other techniques.

The findings are reported as essential data in Table 1 as well as Additional file 2: Tables S1 to S6, which comprised the following information: name of the pathogen in the original article, name of the primer(s) and target DNA, variants utilized in the PCR protocol, type of sample, and treatment prior to amplification, and summarized below according to each pathogen.

### Xanthomonas fragariae

The bacterium *X. fragariae* Kennedy *et* King (Additional file 2: Table S1), the causal agent of angular leaf spot, is a pathogen that spreads in all major areas of strawberry cultivation [16]. It is a very slow-growing bacterium in culture and is easily overgrown by saprophytic bacteria; selective media are not yet available. Therefore, isolation plating is not recommended for the detection of low *X. fragariae* numbers in symptomless plants [11]. Several PCR detection methods each targeting different loci in the *X. fragariae* genome have been developed. Conventional PCR using species-specific primers is known to differentiate close species and used for detection of *X. fragariae* [*S#1,5,6*]. Nested PCR [*S#2,4,7*] is also another main technique used for *X. fragariae*, while multiplex PCR (mPCR) [*S#3*] is found just in one study for detection of *X. fragariae* in plant tissue. Notably, rtPCR assays are used for many bacteria including the species on which we focused in this review, although TaqMan chemistry is prominently used for detection and quantification of *X. fragariae* [*S#8–10*] that could even detect ten bacterial cells in strawberry crown tissue. The detection of *X. fragariae* in crown tissue extract was possible with real-time PCR but not with standard PCR, which is a significant improvement over standard PCR [17]. The assay offers a new tool for epidemiological research and for sanitary control of plant material with low level or latent infections of pathogen [18].

### Phytophthora fragariae

Conventional PCR [*S#16–18*] assays have been developed for *P. fragariae* (Additional file 2: Table S2) targeting different single-copy genes and rDNA spacer region, although studies show contradicting results on detection sensitivity. In this respect, species-specific polymorphisms were exploited in *RAS*-like and *TRP1* genes to develop a set of two *P. fragariae*-specific PCR primer pairs [19]. Thus, it seems to be equally or even more sensitive than other published single-round PCR tests. Real-time PCR and nested rtPCR (nrtPCR) methods using fluorescent-labeled probes (TaqMan™ and Molecular Beacon™) have the necessary properties to fulfill the requirements of an effective detection system and are the other studied procedures for *P. fragariae* diagnostic [*S#13–15*]. The nested PCR-based method is described to be 1,000–10,000 times more sensitive compared to single-round PCR [13]. With Molecular Beacon™ probes, the pathogen is detected in a quantitative order similarly to TaqMan™ probes, which are able to detect 0.1 fg DNA of the pathogen. In a comparative study, the sensitivity of Molecular Beacon™ and TaqMan™ probes against a dilution series of *P. fragariae* genomic DNA was equivalent [13]. Nested PCR is also reported to successfully detect *P. fragariae* [*S#11*] in naturally infected strawberry tissues. Another less investigated test, like PCR-ELISA [*S#12*], has been advised for use in pathogen detection. However, this test cannot be recommended for critical diagnosis, since the sensitivity is comparable to gel electrophoresis and ethidium bromide gel staining [13].

### Botrytis cinerea

*B. cinerea* Pers. Fr. (Additional file 2: Table S3), the causal agent of gray mold or Botrytis blight, establishes symptomless infections, where the pathogen remains latent until the strawberry ripens [20]. TaqMan chemistry [*S#20–22*] based on primers and probes designed to the *b-tubulin* gene, the intergenic spacer (IGS) region of the rDNA, and the species-specific sequence-characterized amplified region (SCAR) as the main genomic regions used to design rtPCR assay was applied for the detection and quantification of the fungus in infected strawberry plant tissue before and after symptom expression [20]. Based on Suarez et al. [21] results, the IGS assay gave a *C*_t_ value <40 of pure *B. cinerea* DNA that was 100 times more sensitive than the SCAR assay and 1,000 times more sensitive than the *b-tubulin* assay. Random amplified polymorphic DNA (RAPD) with Southern blot hybridization [*S#19*] is also an applicable and powerful tool for diagnosis of *B. cinerea* in symptomless strawberry under field conditions [22]. In other words, hybridization of southern blots with RAPD and *Eco*RI-digested DNA confirmed the specificity of the marker for detection and quantification of the pathogen during the latency period. The procedure was able to amplify the 0.7-kb *B. cinerea* fragment from mixed samples of DNA as low as 2 pg *B. cinerea* genomic DNA and 1 μg plant DNA.

### Fusarium oxysporum

*F. oxysporum* f.sp. *fragariae* Winks & Y.N. Williams (Additional file 2: Table S4) is a polyphagous soilborne facultative pathogen causing strawberry wilt disease that has dramatically decreased the commercial production of strawberry [23]. Multiplex PCR [*S****#****23*] was used as the main detection technique to determine the pathogen based on DNA fragments. Although mPCR is becoming a rapid and convenient screening assay for most *Fusarium* spp., it has been used for detection of *F. oxysporum* in strawberry in just one study. Suga et al. [1] characterized and used some transposable elements (sequence-characterized amplified regions) in the pathogen to design a specific set of PCR primers, as shown in Additional file 2: Table S4. The genomic region between *Han* and *Skippy* (as transposable elements) was amplified by an inter-retrotransposon-amplified polymorphism technique (IRAP-PCR), and specific primers were designed from this region. The developed PCR primers discriminated *F. oxysporum* f.sp. *fragariae* strains from nonpathogenic *F. oxysporum* strains and five other *formae speciales* [1]. Use of other PCR-based techniques could not be found for strawberry fusarium wilt diagnosis, while the molecular detection of *F. oxysporum* on other hosts has been reported [24, 25].

### Colletotrichum acutatum

*C. acutatum* (Additional file 2: Table S5) is one of the most frequently reported species of the genus and causes anthracnose disease which is especially destructive on strawberry [26]. For rapid and specific assessment of the pathogen in strawberry, TaqMan rtPCR [*S#27,28*] using primers designed to the rDNA ITS is used and strongly recommended. Development of the *b-tubulin*-based rtPCR primers is less complicated, but the single copy nature of this target leads to primers that are less sensitive and therefore less suitable for detection of *C. acutatum* than the multi-copy ITS regions [27]. In a similar study, Garrido et al. [28] demonstrated that TaqMan rtPCR is 10–100 times more sensitive than cPCR [*S#29*] for diagnosis of the strawberry anthracnose agent. Moreover, nPCR [*S#25*] is another technique which can be successfully used to detect *C. acutatum* on symptomless strawberry leaves. Because of strong detection sensitivity, this method can be applied as a powerful tool for a reliable diagnosis of the pathogen in the field [29]. Since rtPCR is usually less affected by inhibitors such as chlorogenic acid than cPCR [30], detection of *C. acutatum* in necrotic leaf tissue may be more difficult with conventional or nested PCR than with rtPCR.

### Verticillium dahlia

Verticillium wilt, caused by the soilborne fungus *V. dahliae* (Additional file 2: Table S6), is an economically important disease worldwide, which can cause significant crop loss on strawberry even with low soil inoculum densities [31, 32]. Nested amplification [*S#30*] assay and cPCR [*S#31*], using modified DNA extraction methods for amplification improvement, are described as potential assays for detection of *V. dahliae* in the strawberry plant and soil. A multiplexed TaqMan rtPCR [*S#32*] based on the rDNA IGS provides a more specific quantification of the pathogen at low inoculum densities with a higher level of sensitivity. According to Bilodeau et al. [33], the sensitivity of the method allows specific detection of one to two microsclerotia/g of *V. dahliae* in soil, which represents a higher sensitivity compared to other methods for this pathogen.

### Macrophomina phaseolina

*M. phaseolina* (Tassi) Goid., the cause of charcoal rot on strawberry, has a wide geographic distribution because it infects the roots and lower stem of over 500 plant species [34, 35]. Many PCR-based detection methods are able to detect *M. phaseolina* on plant tissues and soil [36–38], but no study has been found for detection and quantification of the pathogen on strawberry. However, published protocols may provide useful information about application of available detection methods on strawberry. Recently, a rtPCR using TaqMan and SYBR Green was published which enables a specific quantification of *M. phaseolina* abundance in rhizosphere and plant tissues [39]. Thus, this method seems to be a strong diagnostic tool for *M. phaseolina*.

## Discussion

A variety of molecular methods have been described for specific detection and identification of phytopathogenic fungi and bacteria. However, the present compilation focused solely on the PCR-based protocols available for routine diagnosis including detection or quantification of strawberry pathogens. Our systematic review identified ten different protocols for *X. fragariae*, eight for *P. fragariae*, four for *B. cinerea*, six for *C. acutatum*, three for *V. dahliae*, and only one for *F. oxysporum*. No PCR-based detection method for *M. phaseolina* in strawberry could be identified in the literature yet. The strawberry pathogens included above were chosen due to their economic impact on crop losses, their distribution, and their status as quarantine organisms. Specificity and sensitivity of methods were identified by systematically summarizing the available literature (Additional file 2: Tables S1 to S6).

Generally, the sequences and the genomic targets of conserved universal genes with enough sequence variation between species are the best choice for designing PCR diagnostic assays. Depending on the genomic region chosen to design PCR primer sets, highly specific diagnostic tests can be obtained, allowing detection of the specific pathogen species and strains from related species or within the same species, respectively [9, 40]. Primer design requires knowledge of the target DNA sequences, and multiple strategies are therefore being developed to design primers for specific detection and disease diagnosis [41–44]. The rDNA operon has frequently been used to design primers that allow highly sensitive detection, but due to its universal nature, the level of discrimination lies at the species levels [15]. The ITS region within prokaryotic and eukaryotic rDNA operons has been described as a stable genetic marker and was used to design primers by Bonants et al. [13, 45], Vandroemme et al. [18], Turechek et al. [17], Kuchta et al. [46], Debode et al. [27], and Garrido et al. [28], among others. Thus, the ITS region is the most widely sequenced for strawberry pathogens. Another genomic portion of the rDNA cistron is the spacer between IGS or the non-transcribed spacer (NTS) that was used to design primers by Suarez et al. [21] and Bilodeau et al. [33]. IGS sequences are more difficult for amplification and sequencing, but they can be more variable than the ITS sequences. Thus, they are exploited to design diagnostic assays when there are not enough differences available across the ITS [9]. Moreover, among conserved genes, the *b-tubulin* has been used to develop diagnostic PCR assay for *B. cinerea* and *C. acutatum*. The sequence of this gene can be useful when the ITS sequence does not allow to fulfill specificity requirements of a diagnostic test [9]. A few loci suitable for the design of species-specific primers for *X. fragariae* have been identified: RAPD-specific regions [47, 48], within the *hrp* [16, 49, 50] and *gyrB* [18, 51] genes.

There is a discussion between results of studies that can be described by four variables. Firstly, in the primers reported here, sequences from pathogenicity-related genes of different species have been employed, although pathogenicity genes are not known in most cases. But, there is an example of the need to design new primers after the discovery of *forma specialis* that lack some pathogenicity genes, previously considered universal. Many types of transposable elements such as *Hop*, *Hornet1*, *Foxy*, *Fofra*, and *Skippy* were considered as excellent targets for *F. oxysporum* f.sp. *fragariae* detection, and several sets of primers were designed on its sequence [1, 52–54]. However, the discovery of nonpathogenic *F. oxysporum* showed that these primers were not as specific as expected [1]. Secondly, the frequent presence of PCR inhibitors in the plant tissues or soil can considerably reduce the sensitivity of the reaction. Thus, a low copy number of initial target DNA sequences makes the first amplification cycles critical, because it may result in false-negative results caused by PCR inhibitors [14]. This could have a major impact on the result of diagnostic tests and therefore is a confounding, but important, variable. In this context, sample preparation is critical, and target DNA should be made as available as possible for amplification. So, an increasing number of commercial kits and DNA extraction protocols for DNA purification and removal of PCR inhibitors from plant materials and soils are available and reported in Additional file 2: Tables S1 to S6. Also, in two included studies [33, 51], internal PCR controls were employed in order to improve sensitivity and avoid false negatives. Thirdly, some results were obtained from nucleic acids present in the soil rather than living cells. Hence, there is a risk of detecting target DNA from dead sources; DNA can persist in soil for long periods of time by forming complexes with soil components and may lead to positive PCR results [55, 56]. Lastly, the majority of the included studies in the literature review investigated PCR methods based on agarose gels for detection/identification of strawberry pathogens. However, some studies focused on quantification of the pathogen using the rtPCR technique, in which sensitivity was increased. Conventional and real-time PCRs are difficult to compare because of the different throughput, sensitivity, and resolution levels [57].

Several methods have been developed to improve sensitivity of cPCR with regard to the goal of this study. Nested PCR with both internal and external primers to the target sequence was reported to increase detection sensitivity and reduce the effect of PCR inhibitors [46, 48, 50]. In fact, when the pathogen is present in very low levels, a higher level of specificity is needed, or the infestations need to be detected in complex environmental samples [50], affecting the reaction. However, the risk of false positives due to cross-contamination of reaction mixtures in routine analysis of large numbers of samples is increased by the introduction of a second round of amplification and the simultaneous manipulation of the previously amplified products [49]. To avoid these problems, nPCR in a single closed tube has been developed [58, 59].

mPCR, a PCR variant which is designed to amplify multiple targets by using multiple primer sets in the same reaction, was applied for detection of *F. oxysporum* in strawberry [1]. Although mPCR consists of a simultaneous screening method in a single reaction tube for the rapid and sensitive detection of different DNA targets, it requires a tedious and time-consuming optimization processes to keep up sensitivity of the single PCR due to competition between different amplification products in one tube [60]. Decrease in sensitivity and limited number of targets that can be simultaneously detected are the most significant drawbacks of mPCR [61]. rtPCR offers better multiplexing possibilities, but multiplexing is still limited by the availability of dyes emitting fluorescence at different wavelengths [14]. A similar limitation to the use of multiplex rtPCR is the competition between different primers and probes which can result in lower sensitivity and specificity [62, 63]. However, Bilodeau et al. [33] reported a rapid and specific determination of soil inoculum densities of *V. dahliae* in strawberry fields without reduction of sensitivity against single amplifications using multiplexed TaqMan rtPCR. Another powerful and practical technique for simultaneous detection of multiple plant pathogens in a wide range of environmental samples is the macro- and micro-array [8, 64, 65], but is not found for the aforementioned pathogens during our literature survey.

In plant disease management, the assumption that rtPCR is more sensitive than cPCR is widely accepted. The higher sensitivity of rtPCR compared to cPCR is determined by two main features: firstly, data are available in real time, are on-screen, do not require time-consuming post-PCR processing, and can be quantitative. Secondly, rtPCR assays commonly amplify very short DNA fragments (70–100 bp) which favors a higher level of PCR efficiency and sensitivity compared to cPCR [28, 66]. In this regard, many different systems have been developed, including probe-based methods, such as TaqMan probes [9, 13, 15] and molecular beacons [13]. In general, the protocols developed are based on hybridization of the probe to the target amplicon, thus achieving maximum sensitivity and confirming the identity of the amplified product [14, 15, 28]. Only 12 kinds of rtPCR protocols are referred to here for detection and quantification of strawberry pathogens. But one should bear in mind that their number has increased from only one in 2004 [13] to six between 2007 and 2012 [17, 18, 27, 28, 33, 51]. It seems that, since amplicon detection through the specific fluorescent signal removes the requirements for post-amplification phases needed in cPCR, it reduces time and considerably promotes the throughput of rtPCR assays, making it suitable for large-scale analyses of the mentioned pathogens. Besides, primers designed for cPCR can be utilized in rtPCR assays if amplicon size criteria are met [57]. Hence, existing cPCR protocols for the detection of plant pathogens can be adapted to be used in real-time detection (rtPCR assays), which can result in a higher level of sensitivity.

In this compilation, all of the rtPCR protocols have utilized probe-based methods, which provide greater sensitivity and specificity than other PCR techniques for detection of strawberry pathogens. However, DNA-intercalating dyes can offer a valid alternative to probe-based methods that bind to double-stranded DNA. SYBR Green is one of the most widely used intercalating DNA dyes for rtPCR applications because of cost efficiency, generic detection of amplified DNA, and its ability to differentiate PCR products by melting curve analysis [67]. Nevertheless, the drawback of using SYBR Green for melting curve analysis is that the melting temperature is highly dependent on the concentration of the dye [68] and DNA [69]. TaqMan™ and SYBR™ green techniques are most widely used for diagnostic purposes, but several considerations must be taken into account. First of all, the TaqMan technique tends to be more specific than SYBR Green due to the use of the sequence specific probe; however, this leads to higher initial costs [70]. Secondly, the SYBR Green method is cheaper to establish since fluorescent-labeled probes are not used; however, SYBR Green fluorophores can also associate with non-specific reaction products such as primer-dimers which may result in poor specificity and false-positive results [57]. Thirdly, availability of instrumentation, the degree of diversity among target and non-target sequences, and the need for multiplexing are primary factors in the choice of real-time platforms [70, 71]. In fact, SYBR Green™ does not allow to multiplex different amplification products. In this scenario, no SYBR Green protocol has been published for detection of studied strawberry pathogens yet. In this review, we also included studies using PCR-ELISA [13] and cPCR using PCR kit [16] that allow detection of *P. fragariae* and *X. fragariae* in strawberry plants, respectively. These techniques are not as powerful as rtPCR in detecting and quantifying pathogens but were nevertheless included in this review since they still did allow a reliable detection of strawberry pathogens.

### Limitations

This review focuses mostly on sensitivity of PCR diagnostic methods outlined in the selected articles. Sensitivity of diagnostic methods is reported in Additional file 2: Tables S1 to S6; however, most studies did not include information on sensitivity levels of the investigated technique(s). Hence, accuracy of measurements based on their performance in pathogen detection was difficult to define because different studies employed different procedures for inoculum preparation, DNA extraction [72–74], and primer design (from different regions of the gene). These will impede to define common pre-analytical requirements, DNA isolation, and amplification procedure (as the factors that affect the efficiency of the test formats) to be employed in routine analyses with the aim of establishing a common diagnostic PCR-based method. Therefore, it was impossible to make direct comparisons between studies. Also, PCR-based quantification of genes amplified from nucleic acids isolated from environmental samples is influenced by a number of confounding factors. Firstly, nucleic acid extraction efficiencies are different between different methods, and so the performance of the final nucleic acid is dependent on both the method used and the type of environmental sample. Most included studies used commercial kits to extract DNA from strawberry tissues, because of their simplicity and rapidity together with the absence of harmful chemical compounds. However, DNA isolation kits can be expensive and inefficient when handling plants with high polyphenolic content. Secondly, many different extraction procedures are used for various samples and within different laboratories, making direct comparison between studies extremely problematic. Therefore, in order to compare detection sensitivity from different environmental samples, it must first be ensured that the same extraction procedure is used for each sample. Indeed, the absence of common pre-analytical procedures might affect final results. Generally, real-time PCR was mostly used in the studies under investigations but not with the same primer and probe, resulting in a restricted comparability. However, while keeping the limitations of the used PCR-based methods in mind, rtPCR remains the gold standard technique for detection and quantification of strawberry pathogens.

## Conclusions

From a systematic review of the currently available published literature, rtPCR is shown to be a particularly promising technique for diagnosing and quantifying pathogen populations in strawberry, whereas some other techniques are suitable for the identification/detection of the aforementioned pathogens. The technique rtPCR allows a specific, reliable, and high-throughput detection of target DNA in symptomless strawberry leaves and various environmental samples in real time. However, we hypothesize that a large proportion, possibly a majority, of errors in laboratories occurs in the pre-analytical phase of the testing process. Therefore, pre-analytical factors need to be considered when applying a diagnostic test. As more PCR-based methods for detection of plant pathogenic fungi and bacteria become available, their use will progressively increase not only for identification purposes but also for different applications, such as studies on pathogen population in their ecosystem in order to facilitate reliable detection. These studies are fundamental to obtain a comprehensive understanding of the pathogen biology with the final intent of optimizing plant disease management strategies.

## Electronic supplementary material

Additional file 1:Search strategy: The file contains a sample search strategy. (DOC 83 KB)

Additional file 2: **Supporting information.** Summaries of the included studies are reported along with the quantitative measurements undertaken by the original articles that assessed detection sensitivity in each protocol. **Table S1**: PCR-based techniques applied for detection of *X. fragariae* in strawberry. **Table S2**: PCR-based techniques applied for detection of *P. fragariae* in strawberry. **Table S3**: PCR-based techniques applied for detection of *B. cinerea* in strawberry. **Table S4**: PCR-based techniques applied for detection of *F. oxysporum* f.sp. *fragariae* in strawberry. **Table S5**: PCR-based techniques applied for detection of *C. acutatum* in strawberry. **Table S6**: PCR-based techniques applied for detection of *V. dahliae* in strawberry. (DOC 139 KB)
