# Supplementary material for: PCR-based specific techniques used for detecting the most important pathogens on strawberry: a systematic review
Source: Syst Rev. 2015 Jan 15;4(1):9. doi: 10.1186/2046-4053-4-9 (PMC4320524; doi:10.1186/2046-4053-4-9)
Supplement: Supplementary file 1 — Additional file 1:Search strategy: The file contains a sample search strategy. (DOC 83 KB) [file 13643_2014_324_MOESM1_ESM.doc]

Search Strategy

Additional file 1

**PCR-based specific techniques used for detecting the most important pathogens on strawberry: a systematic review**

Seyed Mahyar Mirmajlessi^1*^ Email: [m.mirmajlessi@gmail.com](mailto:m.mirmajlessi@gmail.com)

Marialaura Destefanis^2^ Email: [maria.destefanis@agriculture.gov.ie](mailto:maria.destefanis@agriculture.gov.ie)

Richard Alexander Gottsberger^3^ Email: [richard.gottsberger@ages.at](mailto:richard.gottsberger@ages.at)

Marika Mand^4^ Email: [marika.mand@emu.ee](mailto:marika.mand@emu.ee)

Evelin Loit^1^ Email: [evelin.loit@emu.ee](mailto:evelin.loit@emu.ee)

^1^Estonian University of Life Sciences, Institute of Agricultural and Environmental Sciences, Department of Field Crops and Grassland Husbandry, Tartu, Estonia

^2^Department of Agriculture, Food and the Marine, Pesticides, Plant Health & Seed Testing Laboratories, Backweston Campus, Celbridge, Co. Kildare, Ireland

^3^Austrian Agency for Health and Food Safety (AGES), Institute for Sustainable Plant Production, Department for Molecular Diagnostics of Plant Diseases, Vienna, Austria

^4^Estonian University of Life Sciences; Estonian University of Life Sciences, Institute of Agricultural and Environmental Sciences, Department of Plant Protection, Tartu, Estonia

^*^Corresponding author: Estonian University of Life Sciences, Institute of Agricultural and Environmental Sciences, Department of Field Crops and Grassland Husbandry, Tartu, Estonia

**Appendix 1 Sample Search Strategy**

| **Sample Search Strategy [repeated for all databases]** | |
| --- | --- |
| **Search terms** | **No of records returned** |
| ***For AGRIS until 01/04/2014*** |  |
| 1. Polymerase chain reaction | 105,653 |
| 1. PCR | 50,219 |
| 1. PCR method | 200,392 |
| 1. PCR methods | 234,505 |
| 1. Diagnosis | 29,251 |
| 1. Molecular diagnostic | 200,751 |
| 1. “Molecular diagnostic” | 250 |
| 1. Diagnostic, Molecular | 87 |
| 1. Pathogen detection | 94,567 |
| 1. Strawberry | 8,420 |
| 1. *Fragaria ananassa* | 365 |
| 1. Strawberry diseases | 212,726 |
| 1. Diseases, Strawberry | 212,726 |
| 1. "Strawberry diseases" | 24 |
| 1. [#1 or #2 or #3 or #4 or #5 or #6 or #7 or #8 or #9 or #10 or #11 or #12 or #13 or #14] | 363,143 |
| 1. Plant pathogens | 806,856 |
| 1. Plant fungi | 827,677 |
| 1. Plant bacteria | 842,847 |
| 1. Pathogenic fungi | 107,961 |
| 1. Pathogenic bacteria | 114,406 |
| 1. Fungal diseases | 29,062 |
| 1. Bacterial diseases | 255,621 |
| 1. *Fusarium* spp. | 50,714 |
| 1. *F. oxysporum* | 17,942 |
| 1. *“F. oxysporum* f.sp. *fragariae”* | 507,696 |
| 1. *Phytophthora* spp*.* | 86,257 |
| 1. *“P. fragariae”* | 10,243 |
| 1. *Colletotrichum* spp. | 81,081 |
| 1. *“C.* *acutatum”* | 4,602 |
| 1. *Verticillium* sp. | 80,701 |
| 1. “*V. dahliae*” | 4,062 |
| 1. *Botrytis* sp. | 83,078 |
| 1. “*B. cinerea*” | 7,258 |
| 1. *Macrophomina* sp. | 77,887 |
| 1. *“M. phaseolina”* | 1,039 |
| 1. *Xanthomonas* spp. | 81,669 |
| 1. “*X. fragariae*” | 5,429 |
| 1. [#16 or #17 or #18 or #19 or #20 or #21 or #22 or #23 or #24 or #25 or #26 or #27 or #28 or #29 or #30 or #31 or #32 or #33 or #34 or #35 or #36 or #37] | 457857 |
| 1. [#15 AND #38] | 2376 |
| 1. Detection, PCR | 104,131 |
| 1. PCR identification | 117,049 |
| 1. PCR quantification | 60,918 |
| 1. Identification, Strawberry pathogens | 116,075 |
| 1. Detection, Strawberry pathogens | 104,285 |
| 1. Quantification, Strawberry pathogens | 55,519 |
| 1. Strawberry pathogens, PCR | 90,459 |
| 1. *“F. oxysporum* f.sp. *fragariae”,* Detection | 64,775 |
| 1. *“P. fragariae”,* Detection | 771,284 |
| 1. *“C.* *acutatum”,* Detection | 843,423 |
| 1. “*V. dahliae*”, Detection | 394,657 |
| 1. “*B. cinerea*”, Detection | 601,013 |
| 1. *“M. phaseolina”,* Detection | 82,013 |
| 1. *“X. fragariae”,* Detection | 166,661 |
| 1. [#39 or #40 or #41 or #42 or #43 or #44 or #45 or #46 or #47 or #48 or #49 or #50 or #51 or #52 or #53] | 125642 |
| 1. [#15 AND #38 AND #54] | 524 |
| 1. “PCR requirements” | 6,575 |
| 1. Requirements, PCR | 86,093 |
| 1. Analytical | 33,652 |
| 1. Analytical methods | 8,510 |
| 1. Preanalytic | 6 |
| 1. Preanalytical requirement | 14,934 |
| 1. Preanalytical requirements | 56,736 |
| 1. Preanalytical requirements, PCR | 6,575 |
| 1. [#56 or #57 or #58 or #59 or #60 or #61 or #62] | 425642 |
| 1. [#15 AND #38 AND #54 AND #64] | 315 |
