# Supplementary material for: PCR-based specific techniques used for detecting the most important pathogens on strawberry: a systematic review
Source: Syst Rev. 2015 Jan 15;4(1):9. doi: 10.1186/2046-4053-4-9 (PMC4320524; doi:10.1186/2046-4053-4-9)
Supplement: Supplementary file 2 — Additional file 2: Supporting information. Summaries of the included studies are reported along with the quantitative measurements undertaken by the original articles that assessed detection sensitivity in each protocol. Table S1: PCR-based techniques applied for detection of X. fragariae in strawberry. Table S2: PCR-based techniques applied for detection of P. fragariae in strawberry. Table S3: PCR-based techniques applied for detection of B. cinerea in strawberry. Table S4: PCR-based techniques applied for detection of F. oxysporum f.sp. fragariae in strawberry. Table S5: PCR-based techniques applied for detection of C. acutatum in strawberry. Table S6: PCR-based techniques applied for detection of V. dahliae in strawberry. (DOC 139 KB) [file 13643_2014_324_MOESM2_ESM.doc]

Supporting Information

Additional file 2

**PCR-based specific techniques used for detecting the most important pathogens on strawberry: a systematic review**

Seyed Mahyar Mirmajlessi^1*^ ^*^ Corresponding author Email: [m.mirmajlessi@gmail.com](mailto:m.mirmajlessi@gmail.com)

Marialaura Destefanis^2^ Email: [maria.destefanis@agriculture.gov.ie](mailto:maria.destefanis@agriculture.gov.ie)

Richard Alexander Gottsberger^3^ Email: [richard.gottsberger@ages.at](mailto:richard.gottsberger@ages.at)

Marika Mand^4^ Email: [marika.mand@emu.ee](mailto:marika.mand@emu.ee)

Evelin Loit^1^ Email: [evelin.loit@emu.ee](mailto:evelin.loit@emu.ee)

^1^Estonian University of Life Sciences, Institute of Agricultural and Environmental Sciences, Department of Field Crops and Grassland Husbandry, Tartu, Estonia

^2^Pesticides, Plant Health and Seed Testing Laboratories, Department of Agriculture, Food and the Marine, Backweston Campus, Celbridge, Co. Kildare, Ireland

^3^Department for Molecular Diagnostics of Plant Diseases, Institute for Sustainable Plant Production, Austrian Agency for Health and Food Safety (AGES), Vienna, Austria

^4^Estonian University of Life Sciences; Estonian University of Life Sciences, Institute of Agricultural and Environmental Sciences, Department of Plant Protection, Tartu, Estonia

**Description and results of included studies** Name of primers and target DNA, sample treatment in the original article, PCR method, detection sensitivity, reference and amplicon size for the different strawberry pathogens. When the detection sensivity of PCR methods reported in articles differs, the measurement units of pathogens are indicated in different styles.

|  | **S1: *Xanthomonas fragariae*** | | | | | |
| --- | --- | --- | --- | --- | --- | --- |
| **#** | **Variant of PCR method** | **Primer name, Target gene** | **Detection sensitivity** (**CFU/mL)/(fg DNA)** | **Amplicon size (bp)** | **Sample (treatment)** | **Reference** |
| **1.** | Conventional | **XF9/XF11** *hrp* gene | 10^4^ cfu | 537 | Bacteria (DNA extraction protocol) | Roberts *et al*., 1996 [49] |
| **2.** | Nested | **XF9/XF11**  (first round) + **XF9/XF12** (second round) *hrp* gene | 18 cfu | 458 | Bacteria (DNA extraction protocol) | Roberts *et al*., 1996 [49] |
| **3.** | Multiplex | **241A/241B 245A/245B 295A/295B**  **Multiplex** (different primer pairs combinations) **241+245, 241+295,** **245+295, 241+245+295** RAPD fragment | 5×10^-2^ | 550 + 300 + 615 | Bacteria (DNA extraction) | Pooler *et al*., 1996 [47] |
| **4.** | Nested | **XF9/XF11**  (first round) + **XF9/XF12**  (second round) *hrp* gene | 2 cfu | 458 | Bacteria, plant (DNA extraction protocol) | Mahuku and Goodwin, 1997 [50] |
| **5.** | Conventional | **JJ9/JJ12** *hrp* gene | 10^3^ cfu | 478 | Bacteria, Plant (DNA extraction protocol, NaOH/HCl method and boiling method) | Zhang and Goodwin, 1997 [74] |
| **6.** | Conventional | **XF10/XF12** *hrp* gene | 10^2^ cfu | 600 < | Plant (modified DNA extraction Kit) | Stöger and Ruppitsch, 2004 [16] |
| **7.** | Nested | **245A/245B** (first round) RAPD fragment  **245.5/245.267** (second round) 245A-245B fragment | 200 | 286 | Bacteria (DNA extraction protocol), Plant (NucleoSpin Plant Kit) | Zimmermann *et al*., 2004 [48] |
| **8.** | Real-time (TaqMan) | **Xf gyrB/Xf gyrB (**primer)  **Xf gyrB-P (**probe) *gyraseB* gene | 10^3^ cfu | NG | Bacteria (DNA extraction kit or the lysis buffer) | Weller *et al*., 2007 [51] |
| **9.** | Real-time (TaqMan) | **Xfr-QPCR-241/Xfr-QPCR-241** *gyrase* B gene  **Xfr-QPCR-241** (Probe) 5′FAM-labelled 3′BHQ-1-labelled (ITS) | 3×10^2^ cfu | 550 | Bacteria (lysis buffer), Plant (DNA extraction Kit) | Vandroemme *et al*., 2008 [18] |
| **10.** | Real-time (TaqMan) | **q241r/ q241f + q295r/ q295f** Genomic DNA  **Probe** 5′FAM-labelled 3′BHQ-1-labelled (ITS) | 10 cfu | 557 + 634 | Bacteria (DNA extraction kit) | Turechek *et al*., 2008 [17] |
| **#** | **S2: *Phytophthora fragariae*** | | | | | |
|  | **Variant of PCR method** | **Primer name, Target gene** | **Detection sensitivity (fg DNA)/(** **zsp^*^)** | **Amplicon size (bp)** | **Sample (treatment)** | **Reference** |
| **11.** | Nested | **P1/P2** (first round) + **DC1/B5**  (second round) ITS region | 20^*^ | 750 | Fungi (DNA extraction protocol/kit), Plant roots (DNA extraction with phenol/ chloroform) | Bonants *et al*., 1997 [45] |
| **12.** | PCR-ELISA | **ITS1/ITS4** primer  **CPB2** capture probe | 10^3^ | NG | Fungi, Water (DNA extraction protocol/kit), Plant roots (DNA extraction with phenol/ chloroform) | Bonants *et al*., 2004 [13] |
| **13.** | Nested real-time (Taq-Man) | **DC6/ITS4** (first round) + **DC1/MP5**  (second round)  **FTAQ1** probe ITS region | 0.1 | 120 | Fungi, Water (DNA extraction protocol/kit), Plant roots (DNA extraction with phenol/ chloroform) | Bonants *et al*., 2004 [13] |
| **14.** | Nested real-time (Mol. Beacon) | **DC6/ITS4** (first round) + **DC1/MP3** (second round)  **MBPfrag** probe ITS region | 0.1 | 120 | Fungi, Water (DNA extraction protocol/kit), Plant roots (DNA extraction with phenol/ chloroform) | Bonants *et al*., 2004 [13] |
| **15.** | Real-time (Taq-Man) | **DC6/ITS4**  primer  **FTAQ1** probe ITS regioon | 10^3^ | 750 | Fungi, Water (DNA extraction protocol/kit), Plant roots (DNA extraction with phenol/ chloroform) | Bonants *et al*., 2004 [13] |
| **16.** | Conventional | **RAS-PFR109h1/ RAS-PFR109h1 + TRP-PFF309a9/ TRPPFF309a9** *TRP* genes | 5×10^2^ | 403 | Fungi (DNA extraction kit) | Ioos *et al*., 2006 [19] |
| **17.** | Conventional | **RAS-PFR109h1/ RAS-PFR109h1 + TRP-PFF309a9/ TRP-PFF309a9** *RAS-*like genes | 10^3^ | 229 | Fungi and Plant (DNA extraction kits) | Ioos *et al*., 2006 [19] |
| **18.** | Conventional | **TW81/AB28** ITS1–5.8S-ITS2 rDNA spacer region | 2×10^3^ | 832 | Fungi and Plant tisuse (DNA extraction protocol, centrifugation) | Drenth *et al., 2006* [72] |
|  | **S3: *Botrytis cinerea*** | | | | | |
| **#** | **Variant of PCR method** | **Primer name, Target gene** | **Detection sensitivity (fg DNA)** | **Amplicon size (bp)** | **Sample (treatment)** | **Reference** |
| **19.** | Conventional (southern blots hybridization) | **C729+/C729-** (unknown)  **pSRC20** Probe | 200 | 750 | Fungi (phenol-chloroform extraction), Plant leaves (DNA extraction protocol and centrifugation) | Rigotti *et al*., 2002 [22] |
| **20.** | Real-time (Taq-Man) | **Bc3R/Bc3F** IGS region  **Bc3P** Probe (FAM-labelled) | 20 | 95 | Fungi (DNA extraction kit), Plant tissue (DNA extraction protocol) | Suarez *et al*., 2005 [21] |
| **21.** | Real-time (Taq-Man) | **Bc2R/Bc2F** SCAR marker  **Bc2P** Probe (FAM-labelled) | NG | 71 | Fungi (DNA extraction kit), Plant tissue (DNA extraction protocol) | Suarez *et al*., 2005 [21] |
| **22.** | Real-time (Taq-Man) | **Bc1R/** **Bc1F** *b-tubulin* gene  **Bc1P** Probe (FAM-labelled) | NG | 86 | Fungi (DNA extraction kit), Plant tissue (DNA extraction protocol) | Suarez *et al*., 2005 [21] |
|  | **S4: *Fusarium oxysporum*** **f.sp. *fragariae*** | | | | | |
| **#** | **Variant of PCR method** | **Primer name, Target gene** | **Detection sensitivity (conidia/g of soil)** | **Amplicon size (bp)** | **Sample (treatment)** | **Reference** |
| **23.** | Multiplex | **FofraF/FofraR + PFO2/PFO3** *Fot3, Han, Hop, Hornet1, Skippy,* 18S rDNA genes | 10^3^ | 239 | Fungi (DNA extraction kit) | Suga *et al*., 2013 [1] |
|  | **S5: *Colletotrichum acutatum*** | | | | | |
| **#** | **Variant of PCR method** | **Primer name, Target gene** | **Detection sensitivity (fg DNA)/(** **conidia/mL)** | **Amplicon size (bp)** | **Sample (treatment)** | **Reference** |
| **24.** | Conventional | **Calnil/ITS4** ITS region | 10^2^ | 490 | Fungi (DNA extraction protocol), Plant leaves (DNA extraction with lyophilization) | Sreenivasaprasad *et al*., 1996 [73] |
| **25.** | Nested | **ITS1-F/ITS4** (first round) + **CaIntz/ITS4** (second round) ITS region | 1 | 490 | Fungi (DNA extraction protocol, NaOH lysis and DNA extraction kit) | Pérez-Hernández *et al.*, 2008 [29] |
| **26.** | Conventional | **ITS1-F/ITS4**  ITS region | 10 | 490 | Fungi (DNA extraction protocol, NaOH lysis and DNA extraction kit) | Pérez-Hernández *et al.*, 2008 [29] |
| **27.** | Real-time (Taq-Man) | **CaITS_F701/** **CaITS_R815**  ITS region  **CaITS_P710** (Probe) 5´6FAM-labeled 3′BHQ-1-labelled (ITS) | 50 | 80 | Fungi (DNA purification kit and NaOH lysis), Plant tissue (DNA extraction protocol) | Debode *et al*., 2009 [27] |
| **28.** | Real-time (Taq-Man) | **ACUT-F1/** **ACUT-R1** ITS region  **ACUT-PB** (Probe) 5´6FAM-labeled 3´TAMRA labeled (ITS) | 10^2^ conidia | NG | Fungi (DNA extraction kit) | Garrido *et al*., 2009 [28] |
| **29.** | Conventional | **CaInt2 /** **ITS4** ITS1–5·8S-ITS2 region | 10^4^ conidia | NG | Fungi (DNA extraction kit) | Garrido *et al*., 2009 [28] |
|  | **S6: *Verticillium dahliae*** | | | | | |
| **#** | **Variant of PCR method** | **Primer name, Target gene** | **Detection sensitivity (fg DNA)** | **Amplicon size (bp)** | **Sample (treatment)** | **Reference** |
| **30.** | Nested | **DVI/DV2** (first round) + **D1/D2** (second round) ITS region | NG | 320 | Fungi and Soil (DNA extraction protocol and centrifugation), Plant (chloroform/ isomyl alcohol and phenol/ chloroform/ isoamyl alcoho) | Kuchta *et al*., 2008 [46] |
| **31.** | Conventional | **VMSPI/VMSP2 + VDSI/VDS2** ITS region | NG | 140 + 540 | Fungi and Soil (DNA extraction protocol and centrifugation), Plant (chloroform/ isomyl alcohol and phenol/ chloroform/ isoamyl alcoho) | Kuchta *et al*., 2008 [46] |
| **32.** | Multiplexed real-time (Taq-Man) | **Vd-F929-947/ Vd-R1076-1094** IGS region  **Vdhrc FAM** (Probe) 5´6FAM-labeled 3′BHQ-1-labelled (ITS)  **Internal control probe** PPF_Probe_543 | 3 | <200 | Fungi and Soil (DNA Spin Kit, the lysis buffer) | Bilodeau *et al*., 2012 [33] |

NG, Not Given; zsp*, Zoospores
